# Evaluation of rock and fluid intermolecular interaction between asphaltene and sand minerals using electrochemical, analytical spectroscopy and microscopy techniques

**DOI:** 10.1038/s41598-024-51196-3

**Published:** 2024-01-05

**Authors:** Jaber Taheri-Shakib, Ali Esfandiarian, Mahyar Rajabi-Kochi, Ezzatallah Kazemzadeh, Mohammad Afkhami Karaei

**Affiliations:** 1 Department of Research and Technology of the Rock and Fluid Reservoirs, Research Institute of Petroleum Industry, Tehran, Iran; 2 Department of Petroleum Engineering, Marvdasht Branch, Islamic Azad University, Marvdasht, Iran; 3 Department of Petroleum Engineering, Amirkabir University of Technology, Tehran, Iran; 4 Faculty of Research and Development in Upstream Petroleum Industry, Research Institute of Petroleum Industry, Tehran, Iran; 5 Department of Petroleum Engineering, Firoozabad Branch, Islamic Azad University, Firoozabad, Iran

**Keywords:** Solid Earth sciences, Chemistry, Energy science and technology, Engineering, Physics

## Abstract

Long-time contact of heavy crude oil with rock leads to an adsorption phenomenon, which causes the rock surface to become oil-wet and appears as a barrier to the fluid flow in the porous media. However precise understanding of how asphaltene fractions influence sand wettability is lacking. The wetness of neat and asphaltene-aged sandstone was calculated using two relative permeability and contact angle methods. Then the molecular interaction between asphaltene and sand minerals was systematically analyzed using Fourier-transform infrared spectroscopy. Furthermore, the zeta potential was representative of electrostatic properties and surface charge alteration of the sand after these phenomena. Scanning electron microscopy with energy-dispersive X-ray (EDX) analysis also showed elemental mapping and dispersion of asphaltene particles on the rock surface. According to contact angle and EDX analyses of asphaltene samples, the contact angle rises from 115° to 141° by an increase in carbon adsorption on the sand surface from 8.23 to 41.56%. Spectroscopy results demonstrated that hydrogen-bonding, π-bonding, and sulfur-containing compounds such as sulfoxide improve asphaltene adsorption onto the sand surface. The higher the aromaticity index and hydrogen potential index of asphaltene, the greater the ability of asphaltene to change wettability. Adsorption of surface active components would make the surface charge of the sand more negative. The presence of nitrogen/sulfur-containing functional groups on the sand surface changed the electrostatic properties, as a sand surface coated with asphaltene would reduce the percentage of metal cations.

## Introduction

Inorganic and organic precipitates in porous media can lead to formation damage and a reduction in permeability and production^1,2^. The formation of inorganic mineral deposits is a function of water content in the reservoir, and their deposition strongly influences permeability due to the decreasing pressure and changing ionic balance of the water^3^. On the other hand, organic deposits mainly consist of wax and asphaltene. Asphaltene particles adsorb on the rock surface in the same terms as Langmuir isotherm and determined based on van der Waals attraction^4^. The use of chemical solvents or modern technologies, such as ultrasonic and nano technologies, are some of the methods used to eliminate and remove these sedimentsin oil and gas reservoirs^5,6^.

Wax can cause flow issues, which are commonly encountered in wells or pipelines^7^. These precipitates are strongly temperature dependent and depend on the properties of the wax such as the carbon number, the shape of the crystals, and their composition^8^, and occasionally completely plug the pipeline path^9^. Asphaltene deposition is a common problem for oil production in reservoirs close to the wellbore area. However, this phenomenon can occur inside the reservoir due to changes in the chemical balance of crude oil, such as the interaction between injected CO_2_ and oil in EOR processes^10–14^. In another word, wax and asphaltene precipitation resulted from going out of the steady-state condition of the system due to changes in physical (temperature, pressure, and flow rate) or chemical parameters (oil composition)^15–20^.

CO_2_ injection can lead to the instability of asphaltene and eventually change the composition of crude oil^21^. Increasing the contact time of crude oil and destabilizing agent can increase the number of asphaltene deposits. It changes the wettability of reservoir rock from water-wetness to oil-wetness^22^. It is very important to study the changes in wettability under different conditions. If sandstone and quartz formations are in a reducing environment, the presence of traces of organic acid in subsurface conditions can affect the alteration wettability of the formation^23–33^. The presence of different acidic components, including stearic acid, lauric acid, hexanoic acid, and lignoceric acid traces in very low concentrations can also cause mica water wettability alteration^34^. Asphaltene deposition depends on oil and asphaltene functional groups, such as anions, cations, and aromatics, due to induced ionic changes^35^. Therefore, any changes in asphaltene compounds can affect the precipitation process or the amount of crude oil. Extensive studies have been conducted on asphaltene inhibitors and affecting processes, including nano-emulsions^36^, solvents^37,38^, microwaves^39^, and ultrasonic^40^. Hydrogen bonds of asphaltene play a key role in changing the viscosity and oil flow^41^. However, it is important to note that asphaltene mainly precipitates in pore structures, therefore the interactions among the asphaltene particles and reservoir rock surfaces are very imperative.

Over the years, scientists have grappled with the complex issue of asphaltene precipitation from crude oil and its subsequent attachment to a variety of minerals^42–46^. In a related study, Syunyaev et al. (2009) explored the process of crude oil asphaltene adsorption on surfaces composed of dolomite, quartz, and mica. They concluded that mica exhibited the highest level of asphaltene adsorption than that of dolomite and quartz^47^. The surface-active properties of asphaltene particles in the sand pack were the main factor in changing the relative permeability and fluid flow regime in porous media^48^. Asphaltene adsorption in porous media depends on fluid flow during dynamic conditions, since the kinetics mechanism of asphaltene is influenced by the flow of fluid on the asphaltene adsorption on the pore structures in the reservoir^49^. Precipitation and deposition of asphaltene particles in pore structures is a function of pressure and crude oil composition, and changes in these two parameters favor the precipitation of asphaltene in porous media^50^. However, the most influential factors for asphaltene adsorption to the sand surface are the bands and polar compounds of asphaltene, which can be affected by any factor and be easily removed from porous media^51^. One method for categorizing these asphaltene fractionations is based on the extracted functional groups from the Fourier transform infrared spectroscopy (FTIR) analysis^52^. Further results have shown that asphaltene fractionation with the basis of adsorption onto sandstone, limestone, dolomite, and calcite has different properties, and depends on the properties of asphaltene such as carbon fractions, electrical charge, composition, bands, as well as reservoir rock properties^53,54^. From another perspective, the electric potential or electro-kinetic potential of the rock, in addition to the size and polarity of the asphaltene particles, determines the asphaltene precipitation in pore structures to some extent^55^. The heterogeneity of the asphaltene mixture has no significant effect on the adsorption of asphaltene particles on the kaolinite, and the contact of kaolinite with different asphaltene mixtures has different results, indicating the need to adapt the surface to asphaltene for adsorption^56^. The adsorption of polar and large oil molecules such as asphaltene on the surface of the pore structures can cause changes in the surface properties, thereby altering the wettability and fluid flow regime^57^. The change in wettability due to asphaltene precipitation depends on the brine properties presented in the porous media, as well as relative permeability^58^. In addition, by measuring the surface relativity change, it is possible to determine the wettability changes caused by asphaltene precipitation in the reservoir rock, which depend on the chemical composition of aging asphaltene and require an understanding of the spatial distribution and magnitude of surface relativity^59^.

Hu et al. (2018) examined how the presence of a water phase affects the adsorption of asphaltene on silica surfaces. They reached the conclusion that the greater the thickness of the adsorbed water film on the surface, the lower the quantity of asphaltene adsorbed. Although, the recent studies show the presence of heteroatoms can significantly enhance the interactions between silica and asphaltenes, contingent upon their location and type^60,61^, some studies defined the presence of nitrogen in the asphaltene structure decreases its tendency for surface adsorption (Reed, 1968). Bai et al. (2019) used molecular dynamics (MD) technique to determine the impact of heteroatoms (such as N, O, and S) on silica/asphaltene interaction. they expressed that the sulfur atoms increase the van der Waals interaction energies by 25%^62^. Their study also investigated the equilibrium desorption conformation and density profile, revealing that the presence of heteroatoms substantially impedes the desorption of asphaltenes from silica due to the enhanced polar interactions. This hindered desorption was further confirmed by the slower detachment of asphaltenes, as evidenced by the analysis of time-dependent interaction energies and centre of mass (COM) distances technique^62^. Therefore, heteroatoms effectively inhibit the desorption of asphaltenes from silica due to the heightened polar interaction. The results of Bai et al. (2019) study also revealed that the specific adsorption configuration of asphaltenes arises from the competition between the interactions of asphaltene with silica and the π–π stacking interactions occurring among the polyaromatic rings within the asphaltene^62^. Furthermore, terminal polar groups, especially carboxyl (COOH), were observed to contribute significantly to the electrostatic interaction, increasing it from − 81 to − 727 kJ/mol^62^. Literature revealed a notable relationship between the nitrogen content of asphaltenes and the quantity of asphaltene that adhered to the adsorbents on both hydrophilic and hydrophobic silica surfaces^63^.

There are several analyses used in the literature for determining the interaction between asphaltene and rock surface, including FTIR, Scanning Electron Microscopy (SEM), Energy Dispersive X-ray Spectroscopy (EDX), UV–Vis spectroscopy, Atomic Force Microscope-Infrared Spectroscopy (AFM-IR), and contact angle measurement technique^64–69^. Mohammadi et al. (2011) utilized UV–Vis spectroscopy analysis to fit the adsorption of asphaltene on kaolin, smectite, fluorite, and Hemahite based on the Langmuir isotherm equation. They presented five different modified Langmuir adsorption isotherm (four linear and one non-linear isotherm models) by estimation of coefficients and using a genetic algorithm (GA). They concluded that GA provides the most accurate fitting plot for the adsorption of asphaltene on these solid surfaces^67^. Mohammadi et al. (2014) optimized the Langmuir constants to obtain a more accurate estimation with a basis of GA and experimental data. They acclaimed that three minerals of kaolin, smectite, and fluorite follow the Langmuir-type isotherms, however, hematite fitted with a multilayer adsorption isotherm^65^. Mousavi et al. (2021) utilized SEM, EDX, FTIR, and contact angle measurement tests to understand the mechanisms of wettability alteration attributed to the adsorption of resin and asphaltene particles in two different carbonate rock types (calcite and dolomite). Their result showed that the concentration of carbon (C) and oxygen (O) increases on both calcite and dolomite rock surfaces after aging with heavy crude oil (Asphaltene = 14.60 wt%, Resins = 16.90 wt%, Aromatic = 39.30 wt%, and Saturate = 29.20 wt%). Moreover, nitrogen (N) and sulfur (S) (heteroatoms) have appeared in the rock structures. They stated that asphaltene has more capability than resin to create polarization interactions for changing the wettability toward oil-wetness^66^. He et al. (2022) studied the adsorption behavior of asphaltene on various types of clay minerals (montmorillonite, chlorite, kaolinite, and illite) and quartz in a heavy oil sandstone reservoir by UV–Vis spectroscopy. They asserted that the affinity of asphaltene for adsorbing on the aforementioned minerals is in order: montmorillonite > chlorite > kaolinite > illite > quartz^69^. Punase et al. (2023) performed several types of experiments, such as SEM, EDX, and porosity measurements to investigate the effect of the presence and absence of clay minerals in the crude oil samples on the interaction and precipitation tendency of asphaltenes. They inferred that the presence of clay minerals in the crude oil increases the adsorption of asphaltene particles on the rock surface due to higher concentrations of aluminum (Al), calcium (Ca), iron (Fe), potassium (K), magnesium (Mg), and silicon (Si) in asphaltene molecular structure^68^. Samouei et al. precipitated asphaltene and wax in porous media and used FTIR and AFM-IR methods to monitor the deposition of organic sediments on the surface of silicate and carbonate rock samples. They expressed a monolayer of organics adhered to the rock surface through ionic or hydrogen bonding. The organic monolayer acts as a hydrophobic film to alter the wettability of rock from water wetness to oil wetness. Furthermore, they defined monovalent or divalent ions can create a bridge between organic sediments and rock surface to bind carboxylate and hydroxyl groups^64^.

In this study, the interactions of four different types of asphaltene deposits and the surface of sandstone rock are investigated at macroscopic and microscopic scales to unveil how the type of asphaltene, as determined by its functional groups, can exert a notable influence on the wettability alteration of sandstone from water-wet to oil-wet conditions. To achieve this, a comprehensive array of analytical techniques were employed. FTIR, zeta potential, SEM, and EDX analyses were utilized to show the rock-asphaltene interactions in porous media in microscopic and molecular scales. Furthermore, contact angle measurements, relative permeability assessments via core flooding experiments, and viscometry were used to show how these interactions change the properties of porous media. This multi-faceted approach allows us to gain a holistic understanding of the asphaltene-sandstone interactions.

Although existing literature has explored the interactions between asphaltene and rock surfaces, this study stands out in its systematic integration of all these commercially relevant analyses at both macroscopic and microscopic scales, and it emphasizes the distinctive impact of asphaltene type on these interactions. It should be mentioned that this study contains different types of analytical techniques, including spectroscopy, electron microscopy, and electrochemistry analyses to obtain more accurate results and support for the hypotheses, a point that is not usually seen in previous studies. Notably, it's worth mentioning that a significant portion of prior research has primarily focused on carbonate rock types, owing to the prevalence of carbonate reservoirs in comparison to sandstone reservoirs.

## Materials and methods

### Rock and fluid samples

Four sandstone core samples were utilized for the experiments, and their properties are shown in Table 1. The selected samples are characterized by similar structures and are free of any fractures, as they have been taken close to each other from a whole core section.

**Table 1:** Petrophysical properties of core samples.

Sample ID	Length (cm)	Diameter (cm)	Porosity (%)	Permeability (md)
1	7.11	3.71	17.26	23.29
2	7.09	3.71	16.94	24.14
3	6.97	3.71	17.31	25.22
4	7.05	3.71	17.11	23.97

Four samples of crude oil from the reservoirs of south-western Iran with the letters A, B, C, and D were used in this study. The characteristics of four crude oil samples including specific gravity (°API), kinematic viscosity (mm^2^ s^−1^) at 37.5 °C, the weight percentage of SARA, total acid number (TAN), and total base number (TBN) are presented in Table 2.

**Table 2:** Properties of crude oil samples.

Sample	API	Viscosity at 37.5 °C (mm^2^ s^−1^)	SARA test				Total acid number (TAN)	Total base number (TBN)
			Saturate (%wt)	Aromatic (%wt)	Resin (%wt)	Asphaltene (%wt)		
A	30.12	10.9	48.18	36.21	3.1	12.51	0.11	< 0.05
B	32.9	10.4	54.68	31.62	2.5	11.2	0.11	< 0.05
C	29.02	11.2	41.28	42.06	3.3	13.36	0.14	< 0.05
D	29.78	10.6	59.54	25.54	2.9	12.02	0.13	< 0.05

The solvents used in this study were toluene (Merck, 99.9% pure) and normal heptane (Merck, 99.9% pure). For irreducible water saturation and measurement of relative permeability, water formation prepared from wells in one of the fields of southwestern Iran was used.

### Asphaltene precipitation procedure

**Figure 1:**
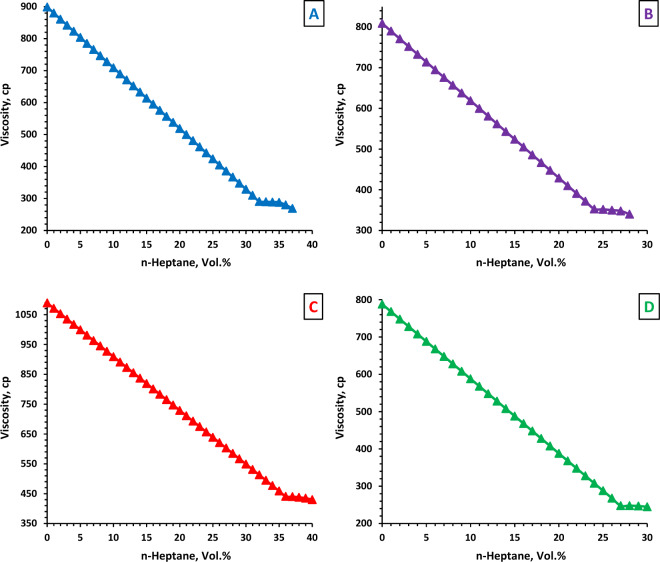
Onset of asphaltene aggregation.

Based on the ISO 3104 and ASTM D445 standards (Fig. 1), a SVM-3000 Stabinger viscometer was used to determine the onset of asphaltene precipitation for different crude oil samples containing varying percentages of n-heptane^70^. The core samples were flooded with a blend of crude oil and n-heptane at 1000 psi overburden pressure and 3.5 cc min^−1^ flow rate (at ambient temperature). Outlet pressure was set at ambient condition and the back pressure regulator did not use. The crude oil and n-heptane mixture is injected at the aforementioned flow rate until the permeability of cores reduces to maximum values^70–72^. According to the preliminary tests, the highest reduction in permeability was achieved at 3.5 cc min^−1^. Fifteen pore volume of oil and n-heptane mixtures were flooded into each core sample.

Table 3 corresponds to the variations of permeability when asphaltene particles are deposited in the core samples. In order to remove the n-heptane from the inside of the core samples, the samples are dried in an oven at 80 °C for 48 h. The core samples are then flooded with toluene to remove the fractions of asphaltene that do not adsorb on the rock surfaces and produce oil under the reservoir conditions^53^. Finally, the adsorbed asphaltene particles will remain in the pore structures after the flushing of bulk asphaltene fractions.

**Table 3:** Variations of permeabilityfor each core sample during different steps of asphaltene deposition.

Core sample	Type of injected asphaltene	Permeability (md)		
		Primary	After whole asphaltene precipitation	After adsorbed asphaltene precipitation
1	A	23.29	4.15	9.73
2	B	24.14	5.42	9.22
3	C	25.22	4.82	7.55
4	D	23.97	5.37	8.11

The remaining asphaltene particles in the reservoir are known as the adsorbed fraction. This fraction is an organic material that is mainly composed of organic fatty acids and is known to be primarily responsible for changing the wettability of rock. The organic fatty acids in the reducing environment improve the interaction between asphaltene particles and the rock surface (carbonate/sandstone/mica) and result in surface adsorption^21,22^. Each core sample is flooded with toluene until the color of the sample becomes entirely transparent and no further asphaltene particle is produced (Table 3).

### Relative permeability measurement

The recycling operation method was used for measuring the steady-state relative permeabilities^73^. Brine with 3% wt sodium chloride (NaCl) was used to obtain relative permeability curves. This concentration was previously selected as the optimal concentration in sandstone experiments to prevent the occurrence of clay swelling. It should also be noted that relative permeability curves were measured under ambient conditions (ambient temperature and pressure).

The relative permeability charts were plotted using the aforementioned basic principles based on the saturation of the wet phase. A BT100S metering pump (with variable pump heads and tubing manufactured by Lead Fluid Technology Co) with a flow range between 0.00011 and 720 cc min^−1^ was used for circulating simultaneously oil and brine at a specific rate. A calibrated capillary flow meter was utilized for each of the injection fluid samples before entering into the core^70^. To form a steady-state condition, the produced fluid samples (oleic and aqueous phases) were collected using a graduated burette and then re-injected from the outlet of the core holder into the core. For evaluating the steady-state conditions, the pressure drop across the core and two flow meters were checked and the interface between aqueous and oleic phases was monitored inside the burette. Moreover, pressure transducers and a computer system were employed for data collection and data logging. It should be noted that two different pressure transducers with varied accuracy were utilized for measuring the pressure drop of each point. For minimizing the pressure fluctuations resulting from dual-piston pumps, a brine surge tank and two pulsation dampeners were used. The relative permeability of each fluid was quickly calculated from the ratio of the pressure drop across the capillary to the pressure drop for each core based on the proportion between pressure drop in a capillary flow, flow rate, and viscosity of the produced fluid. Furthermore, variations in the oleic phase inside the core are observed as changes in the holdup volume in the burette separator, because the total volume of oil saturation is constant during the recycling process. Thus, the oil saturation for each fractional flow can be determined using the mass balance. The initial saturation for each dry core can often be calculated via its history. For the case of reservoir cores, the reference point is measured by extracting a core at the end of the flow test.

### Determining the contact angle

Holmarc’s Contact angle meter Model no: HO-IAD-CAM-01 (based on the sessile drop method) was utilized to determine the contact angle among oil drop and rock surface with the presence of aqueous bank phase at ambient conditions^74–76^. The drop snake method has been used for curve fitting analysis. For conducting each sessile drop experiment, at least four oil drops were thrown on various parts of the rock samples, and then the drops were carefully observed and analyzed. Each drop volume was 6 μL and the mean contact angle was measured for all of the oil drops present in each rock sample. The contact angle is measured 20 min after placing the drop of oil on the rock^77^. The formation brine was synthesized based on the KCl with total dissolved solids (TDS) of 130,000 ppm and the same oil was used to measure the contact angle of the rock sample^10,21–24,34^.

### Scanning electron microscope (SEM) imaging and elemental analysis

SEM images of core samples and adsorbed asphaltene were taken along with mapping, in order to better analyze them. We used a Vario Max-CHNSO elemental analyzer to measure concentrations of C, H, S, N, and O (ASTM D5291–ASTM D4294).

### Fourier transform infrared spectroscopy (FTIR)

A VERTX70 device was used to obtain the FTIR spectrum for different types of samples. The range of each spectrum is from 4000 to 400 cm^−1^, with a resolution of 2 cm^−1^ and 128 scans. The KBr pellet procedure was performed to record the spectrum of asphaltene samples. The dry KBr powder was utilized to disperse each sample (asphaltene and its sub-fractions) and then a pellet was made from the mixture for each asphaltene sample.

### Zeta potential

Zeta potential tests were performed For determining the charge of the rock surface, adsorbed minerals, and asphaltene particles. A blend of minerals and asphaltene adsorbed to the rock surface was prepared (0.1 g) and mixed with 9.9 g of formation brine (KCl = 130,000 ppm)^53^. After sample preparation, the zeta potential was calculated using a zeta sizer manufactured by Malvern Panalytical Co. The temperature range of the device is 0–120 °C and its particle size range is 0.3 nm–10 μm.

Zeta potential is an important indicator of colloidal stability because it measures the ability of particles to resist collision, coalescence, aggregation, and subsequent sedimentation. In principle, dispersions with a zeta potential of less than ± 5 mV are unstable, and ± 10 to ± 30 mV are considered incipient stable. Also, dispersions with zeta potential between ± 30 to ± 40 mV are moderately stable. Dispersions with zeta potential in the range ± 40 to ± 60 mV are considerably stable. Basically, dispersions with zeta potential over ± 60 mV are significantly stable. Dispersions with a zeta potential greater than ± 40 mV are generally known as stable suspensions^78^.

## Results and discussion

### Relative permeability and contact angle

Wettability is the determining factor for the movement of reservoir fluids^73^. When the oil approaches the onset of asphaltene precipitation in porous media, the thermodynamic equilibrium between the asphaltene and the peptides (such as resin) is disrupted and the asphaltene is precipitated on the surface of the rock. This causes the interaction between the surface of the rock and the water phase in the reservoir to be reduced and the system trends towards being neutral or oil-wet^79^. In this case, the oil-wetting state is expected to be strengthened and the production of water cut in the wells will increase over time. In addition, the changes in wettability cause the residual irreducible water saturation to be significantly reduced within the pore space of the rock^72,73,77–80^.

The effect of changing wettability on the fluid's two-phase flow in the reservoir is also observed in the relative permeability endpoints. The relative permeability of the oil phase in connate water saturation [Kro (Swc)] varies, depending on the wettability of the surface. For example, in a strongly water-wet system, its value is considerable, but in a strongly oil-wet system, it will be very low (about 0.2). Furthermore, the higher the crude asphaltene content, the greater the wettability alteration and the lower the amount of irreducible water saturation within the core^81,82^.

**Figure 2:**
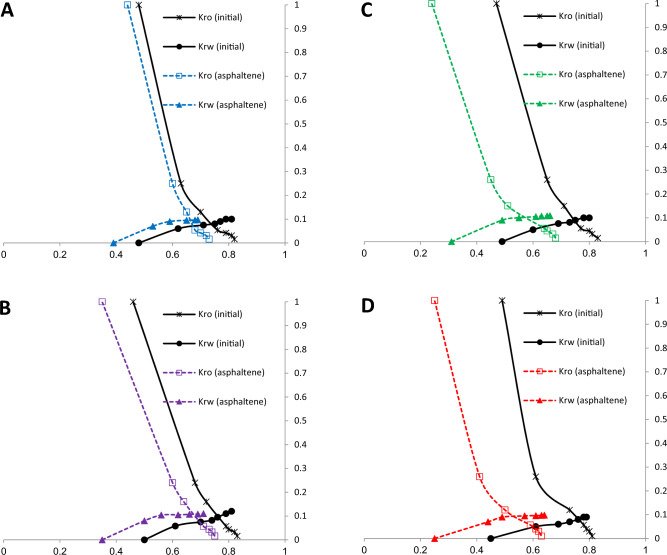
Relative permeability of aqueous and oleic phases in clean sand and after precipitation of four asphaltene samples in the core.

Figure 2 illustrates the relative permeability values of aqueous and oleic phases in terms of wetting phase saturation under “initial” and “asphaltene” states. The “initial” state refers to clean sand with no asphaltene deposition. The “asphaltene” state refers to fluid flow in porous media after asphaltene aging onto the sand surface. Since previous studies have shown that the aging period of asphaltene particles and resin onto a surface can affect the degree of wettability, this parameter was considered for all four samples^83^. In order to compare the wettability of the sand surface due to asphaltene precipitation for each sample, the relative permeability term of each phase rather than absolute rock permeability was used, since asphaltene precipitation has a significantly greater effect on relative permeability compared to absolute permeability^58^.

According to Fig. 2, the system will be oil-wet following asphaltene precipitation in the core, since during the imbibition process, the relative permeability of the oil phase on the "asphaltene" graph is reduced compared to the "initial". The relative permeability of the water phase has also increased in the "asphaltene" state relative to the "initial". This indicates the incremental trend in the amount of gravity in the oleic phase, and eventually, the amount of gravity in the aqueous phase has been reduced. An effective capillary pressure on the front of an imbibition process is created due to the difference among the aforementioned forces and subsequently, prevents the production of a non-wetting phase at the core output. For this reason, it is expected that water breakthrough time will be delayed in oil-wet reservoirs. It should be noted that effective capillary pressure is actually the difference between the pressures that push the wetting phase toward the rock wall and push the non-wetting phase outward^84^.

To quantitatively compare the change in wettability on the sand surface, the crossover point shift can be used. The crossover point is said to have a degree of saturation from the water phase in the porous media, in which the relative permeability of the water and oil phases is equal, and the relative permeation diagrams of the two phases crossover. The crossover point of each graph in both the “Initial” and “Asphaltene” modes is demonstrated in Table 4. The sequence below shows the order of the crossover shift (CS) values after the deposition of each sample of asphaltene:

**Table 4:** Crossover point for clean sand and sand sample after asphaltene precipitation.

Asphaltene Samples	“Initial” Crossover point	“Asphaltene” Crossover point	Crossover Shift
A	0.737	0.665	0.072
B	0.759	0.671	0.088
C	0.747	0.569	0.178
D	0.761	0.528	0.233

1 $${CS}_{A}<{CS}_{B}<{CS}_{C}<{CS}_{D}$$

The crossover point is expected to occur at a lower saturation level than the water phase in the case of an oil-wet sand surface. Therefore, in all samples, the crossover point has been moved to the left of the horizontal axis by adsorbing asphaltene onto the surface of the rock. The adsorption of asphaltene sample D creates the greatest change in wettability. Asphaltene sample C creates less variation in wettability than sample D (crossover shift = 0.178). The two other asphaltene samples, A and B, showed only a small potential for oil-wetting the surface, resulting in smaller changes in surface wettability, with crossover shift values of 0.072 and 0.088, respectively. Samples A and B exhibit lower surface wettability change compared to the other samples.

If the crossover saturation degree of a sand sample is less than 50%, the sample would be strongly oil-wet. Therefore, it can be concluded that asphaltene samples C and D have made the sand surface oil-wet by adsorption^85^.

Residual oil saturation (ROS) was measured before and after the precipitation of each asphaltene sample, and the results are shown in Table 5. After water flooding in water-wet systems, the oleic phase presents as a discontinuous phase in the form of fine droplets within large pore spaces. Meanwhile, in oil-wet systems, continuity is observed in the residual oil phase, which often leads to the establishment of a continuous phase, as oil in these conditions surrounds the rock grains and will be distributed in the form of a thin film around them^86^. According to the results of Table 5, the residual oil saturation shift (ROSS) after the adsorption of four asphaltene samples is in accordance with sequence 2:

**Table 5:** Residual oil saturation (ROS) point for clean sand and sand sample after asphaltene precipitation.

Asphaltene Samples	ROS “Initial”	ROS “Asphaltene”	ROS shift
A	0.18	0.27	0.09
B	0.17	0.25	0.08
C	0.17	0.32	0.15
D	0.19	0.37	0.18

2 $${ROSS}_{A}<{ROSS}_{B}<{ROSS}_{C}<{ROSS}_{D}$$

The results of sequences (1) and (2) are consistent. The adsorption of asphaltene sample D significantly increased the ROS. Sequence (2) predicts that the trend of wettability will shift towards oil-wetting. Some studies have indicated that ROS in water-wet systems is about 20–30%, which is reduced by about 5–10% in an oil-wet system^87^. However, this claim is rejected in this study.

The amount of irreducible water saturation (IWS) was measured before and after the precipitation of asphaltene in the core, and the results are displayed in Table 6. There is a remarkable difference between the values of S_wi_ before and after asphaltene precipitation. Small pores, narrow cracks, and the surface of the grains are occupied by an irreducible aqueous phase in a water-wet system. However, in the oil-wet system, the oleic phase occupies small pores and water only remains on the grain surface. For this reason, IWS decreased in all samples after asphaltene precipitation. Sequence (3) shows the order of the irreducible water saturation shift (IWSS) value, with an increase in this value implying oil-wetting of the sand surface:

**Table 6:** Irreducible water saturation (IWS) point for clean sand and sand sample after asphaltene precipitation.

Asphaltene Samples	IWS “Initial”	IWS “Asphaltene	IWS shift
A	0.48	0.39	0.09
B	0.50	0.35	0.15
C	0.49	0.31	0.18
D	0.45	0.25	0.20

3 $${IWSS}_{A}<{IWSS}_{B}<{IWSS}_{C}<{IWSS}_{D}$$

The above sequence is exactly the same as sequences (1) and (2). Therefore, it is predicted that asphaltene sample D causes the highest change in wettability.

In order to verify the results of the imbibition and drainage relative permeability test, the contact angle of sand samples was prepared after the precipitation of the asphaltene samples. These results are shown in Table 7. The clean sand sample, based on its contact angle, has a neutral surface in terms of wettability. Following the adsorption of asphaltene sample A, the contact angle was 115°, which equates to an oil-wet surface. However, the contact angle after the adsorption of asphaltene sample A has changed less compared to the adsorption of the other samples^88^. Asphaltene adsorption of samples B, C, and D increases the contact angle to 123°, 139°, and 141°, respectively. The changes in contact angle are consistent with the wettability results from the relative permeability diagrams.

**Table 7:** Contact angle values of sand surface after asphaltene precipitation.

Sample	Contact angle
Clean sand	96°
A	115°
B	123°
C	139°
D	141°

On one hand, altering the wettability of the rock surface from a water-wet to an oil-wet state leads to an increase in the relative permeability endpoint for water. This change occurs because the aqueous phase relocates from the surface to the interior of larger pores, allowing for more efficient transport of the aqueous phase through the porous media. Conversely, the relative permeability endpoint for oil decreases as a result of the reduction in water saturation within the porous media, with the oleic phase occupying the rock surface^61,89^. Generally, the modification of rock surface wettability, particularly through the presence of carboxylic acids and other acidic and polar components in crude oil, plays a significant role in this alteration. However, it is worth noting that the wettability alteration due to asphaltene adsorption exhibits a different impact on the endpoint of water relative permeability. When asphaltene particles adhere to the rock surface, not only does the wettability shift towards oil-wetness, but the radius of pore structures also diminishes. This reduction in pore radius results in an increase in the threshold capillary pressure within the pores, hindering the infiltration of non-wet phases (aqueous phase). Consequently, the relative permeability of water decreases as a consequence of wettability modification induced by asphaltene precipitation.

### Characterisation by FTIR spectrometry

#### Whole asphaltene

**Figure 3:**
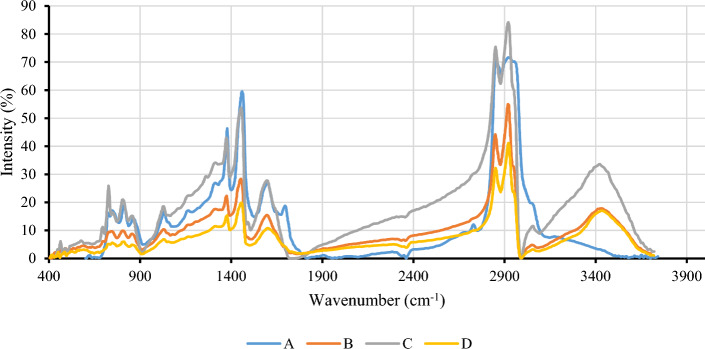
FTIR spectra of four asphaltene samples derived from the IP143 method.

The FTIR range is 400–4000 cm^−1^. Figure 3 shows the spectrum of four asphaltene samples resulting from the IP143 method. In the FTIR spectrum of asphaltene, the frequency range of 2500–3700 cm^−1^ is known as the hydrogen bonding region. Oxygen, nitrogen, and sulfur are the most important heteroatom elements of asphaltene. Oxygen in the form of the carboxylic acid functional group has an O–H bond in its molecular structure. The O–H bond establishes hydrogen bonding with adjacent molecules due to the absence of symmetry in the electron cloud surrounding the bond^53,90^. The corresponding peak of the O–H stretching is normally observed at a frequency range of 3000–3700 cm^−1^ as sharp and wide^26,30,31,91–94^. As shown in Fig. 3, three samples (B, C, and D) have a peak at a frequency of 3400 cm^−1^, but sample A has low intense peak at this frequency. Therefore, asphaltenes in sample A do not have the ability to establish hydrogen bonds.

At a frequency of 3050 ± 10 cm^−1^, a very low intensity is observed that corresponds to the C–H stretch^95^. According to Fig. 3, this peak is observed in the spectrum of all samples and its intensity is less than the peak intervals of 2800–3000 cm^−1^, which is associated with aromatic functional groups. The hydrogen potential index (HPI) is a quantitative indicator that specifies the ability of the molecule to establish a hydrogen bond with adjacent molecules. It is defined as the O–H stretching bond area to the aromatic C–H stretching band^96,97^:

4 $$HPI=\frac{{A}_{O-H\,Stretching}}{{A}_{aromatic\,C-H\,Stretching}}$$

The area of each peak is considered from valley to valley and is identified by the symbol A in Eq. 4. The higher the HPI index of an asphaltene sample, the greater its willingness to establish H-bonding. Table 8 shows the calculated HPI value for each of the four asphaltene samples. The highest tendency for hydrogen bonding among the four asphaltene samples belongs to sample D (HPI = 24.711). In the spectrum of sample C, although the peak corresponding to the O–H bonds stretching has a greater area and is wider than the sample D spectrum, the potential to establish hydrogen bonds is comparatively lower (HPI = 15.079). Therefore, the O–H stretching area cannot be a sign of inter-molecular H-bonding alone.

**Table 8:** The values of calculated quantitative indices for asphaltene samples.

Asphaltene sample	A	B	C	D
HPI	–	1.2853	15.0798	24.711
O–H Stretching Area	–	2506.010	12,058.34	5335.29
Aromaticity Index (AI)	0.182	0.252	0.252	0.305
Substitution-Condensation Index (SCI)	0.8652	1.299	1.4383	1.969
Aliphatic Index (ALI_1_)	5.269	5.138	5.694	6.456
A-factor	0.845	0.798	0.798	0.766

Furthermore, according to the results of Table 8, it was found that asphaltene samples with a higher HPI index would be more capable of changing the wettability of the sand surface. Sequence (5) represents the HPI index of asphaltene, which is quite similar to the sequence of contact angle changes exhibited by the four asphaltene samples:

5 $${HPI}_{A}<{HPI}_{B}<{HPI}_{C}<{HPI}_{D}$$

Therefore, the higher the HPI index in the asphaltene sample, the greater its willingness to alter the wettability of the sand surface towards oil-wetting.

**Figure 4:**
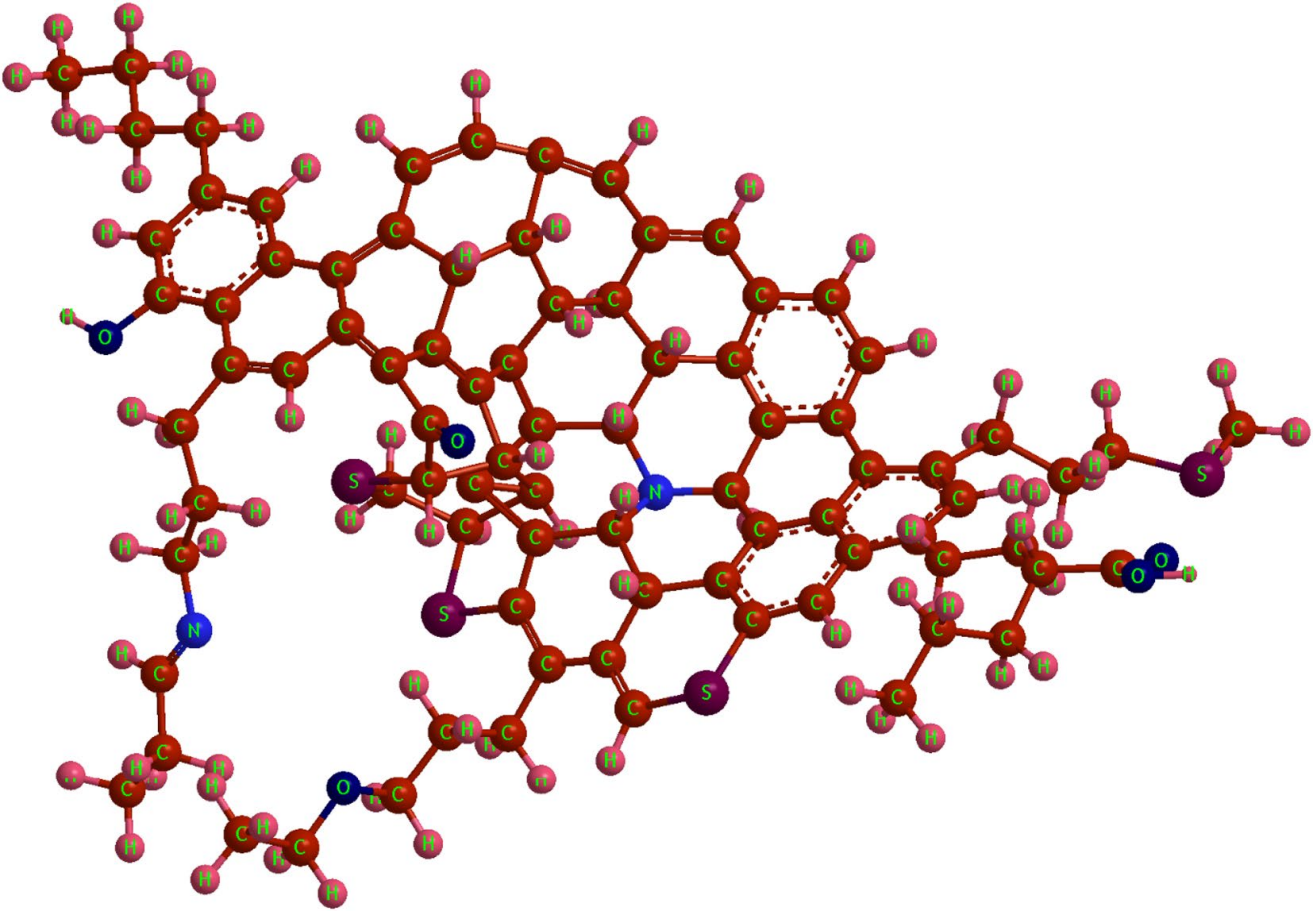
Schematic representation of the asphaltene molecule and its components, consisting of poly-aromatic nuclei, alkane chains, and polar fractions.

The asphaltene molecule has three main parts according to Fig. 4. The first part consists of heteroatom and polar compounds, the second comprises poly-aromatic nuclei which consist of condensed aromatic rings, and the third comprises the side alkyl chain attached to the central aromatic core, which are normally paraffinic chains composed of methyl and methylene groups. Each of these groups plays a unique role in the reactivity of the asphaltene molecule.

Poly-aromatic nuclei appear in the FTIR spectrum via two peak sets. The first is at a frequency of 1600 ± 10 cm^−1^, which corresponds to skeletal vibration involving C–C stretching within the ring, and is present in the spectrum of all samples^98,99^. In some samples such as sample C, the skeleton band appears double, indicating its aromatic nature (existing peaks at 1600 cm^−1^ and 1492 cm^−1^). In the spectrum of poly-nuclear molecules such as asphaltenes, bands are observed in the frequency range 650–900 cm^−1^. These peaks are related to the out-of-plane C–H bending vibration. Compared to the substituted benzene ring, the precise position of these peaks depends on a variety of factors, such as the number of adjacent hydrogen atoms on the fused rings and the hydrogen-substituted functional group^73,77^. Table 9 accurately provides the peak frequencies in this area for four asphaltene samples.

**Table 9:** Frequency of peaks in the range 650–900 cm^−1^ for four samples of asphaltene.

Asphaltene sample	Isolated H (cm^−1^)	Two adjacent Hs (cm^−1^)	Four adjacent Hs (cm^−1^)
A	864	808	750
B	857	806	744
C	857	804	748
D	857	810	747

**Figure 5:**
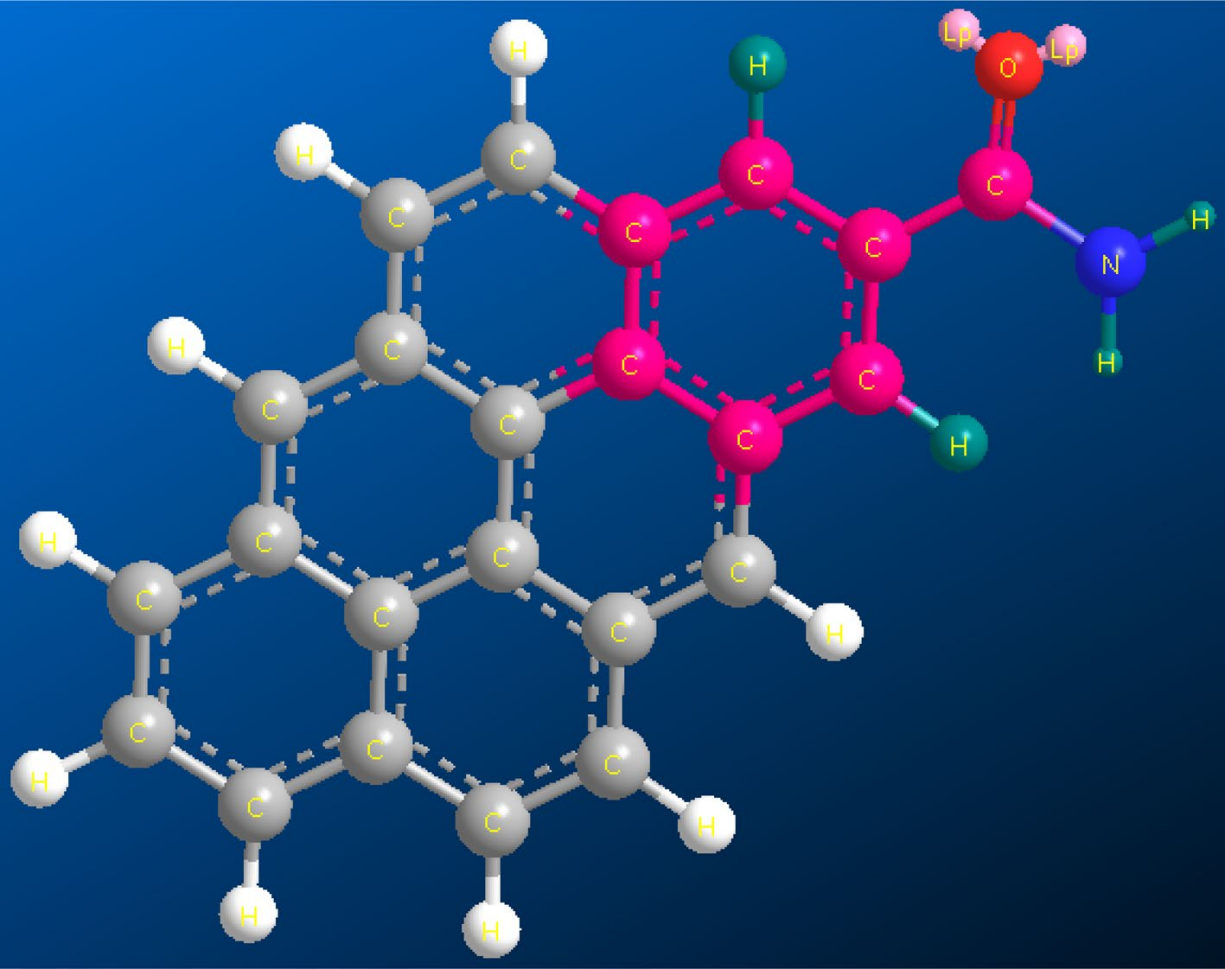
Schematic diagram of the connection of NH_2_ to the carbonyl functional group and the aromatic ring position in sample A.

The corresponding peak of the isolated H appeared in asphaltene sample A at a frequency of 864 cm^−1^. In the three other asphaltene samples, this peak is observed at a frequency of 857 cm^−1^. This is due to the presence of a carbonyl functional group attached to the aromatic ring in sample A. In the spectrum of sample A, a moderate intensity appears at a frequency of about 1700 cm^−1^, which corresponds to C–O stretching in the carbonyl group^53,55–59,74,75,100^. If the electron-donating character is reduced at a substituent, such as a carbonyl group, firstly the appearance frequency of isolated hydrogen increases, and secondly, its intensity will be reduced^101^. Furthermore, the peak corresponding to C–O stretching is observed in a substance that consists only of the carbonyl group at a frequency of 1715 cm^−1^^95^. In the spectrum of sample A, this peak was observed at a frequency of 1695 cm^−1^. Specifically, the peak frequency is about 20 units lower than pure carbonyl, which is mainly due to the connection of nitrogen-containing compounds such as NH_2_ to the carbonyl group and its resonance. According to the literature, the connection of NH_2_ to the carbonyl group causes C–O stretching peaks to appear within the 1650–1695 cm^−1^ frequency interval. Figure 5 shows schematically how NH_2_ is bound to the carbonyl functional group and the aromatic ring position in sample A.

The presence of the hydroxyl group in the molecular structure of the poly-nuclear rings as a substituent causes the four adjacent hydrogens C–H bending vibrations at a frequency of about 748 cm^−1^ to be sharp, and two adjacent hydrogen C–H bending vibrations at a frequency of 807 cm^−1^ to appear medium^101^. The results of Table 9 show that two samples (C and D) are phenolic hydroxyl or carboxylic acid, substituted in the aromatic ring. It has been previously reported that these two samples have the ability to make a hydrogen bond and have a peak at 3400 cm^−1^^101^.

The sharpest peaks of the asphaltene spectrum are in the range of 2800–3000 cm^−1^, which corresponds to C–H stretching. The peaks in this area mostly belong to two major functional groups: (1) methyl with a molecular structure of CH_3_, which is generally observed on both sides of alkane chains. The corresponding peaks of the methyl group were observed at a frequency of 2957 cm^−1^ in the spectrum of sample A, which demonstrates the significant concentration of paraffinic compounds in this asphaltene sample. (2) Methylene with the chemical formula CH_2_, which is present in aromatic and paraffinic structures. The frequency of 2920 ± 10 cm^−1^ is related to the methylene C–H asymmetric stretching bond, and the 2850 ± 10 cm^−1^ peak corresponds to the methylene C–H symmetric stretching bond. These two peaks were observed in the spectrum of all four samples of asphaltene^9,102^.

In the spectrum of all four asphaltene samples in the fingerprint region, there are two other peaks corresponding to paraffinic compounds. The peak at a frequency of 1455 ± 10 cm^−1^ corresponds to the CH_2_ scissoring mode, and the peak at a frequency of 1375 ± 10 cm^−1^ is also assigned to the CH_3_ umbrella vibrating^54,99,101–103^. The methylene functional group present in the asphaltene molecule, in addition to the scissoring vibration, has three other types of vibration: (1) rocking, (2) wagging, and (3) twisting^104^.

The corresponding peak to rocking vibration appears in the FTIR spectrum the amount of methylene in the alkane chain structure is more than seven. This peak is located at a frequency of 724 ± 10 cm^−1^ and is also visible in the spectrum of all samples. Considering this, a quantitative index is used to calculate the carbon-fiber number of the side alkyl chain attached to the aromatic ring and free chains, termed the long chain index (LCI), and calculated from the following formula:

6 $$LCI=\frac{{A}_{724}}{{A}_{1460}+{A}_{1376}}$$

The main component of the alkane chains is methylene, and the longer the chains, the greater the number of methylene attached to the chain. Thus, the intensity of the peak belonging to the methylene group is expected high in the case of long chains^105^. The results of the LCI index for the four asphaltene samples are presented in Table 8. Sample C has the highest index and samples A and D have the lowest values. Therefore, it seems that the length of the carbon-paraffinic branches attached to aromatic rings or free aromatic ring branches does not have a significant effect on altering the wettability of the sand surface. In the frequency range 1150–1350 cm^−1^, samples A, B, and C exhibited very low-intensity peaks and like shoulders for strong peaks at a frequency of 1312 cm^−1^. This peak corresponds to the methylene wagging and twisting modes, which did not appear in sample D^95^.

To evaluate the aromatic and aliphatic properties of asphaltene samples, quantitative indices are used. Equation 7 has been used to compute the aromaticity index (AI) in the literature. The area under the C–H aliphatic stretching adsorption is selected as A_1_ within the interval 2770–3000 cm^−1^, and the region below the C–C aromatic stretching band within the interval 1500–1630 cm^−1^ is selected as A_2_^106,107^.

7 $$AI=\frac{{{\text{A}}}_{2}}{{{\text{A}}}_{1}}$$

The results of the calculated AI for each of the asphaltene samples are shown in Table 8. Sample D has the highest AI among all samples (AI = 0.305). Two samples (B and C) have the same AI values (AI = 0.252), followed by sample A with the lowest AI (AI = 0.182). Therefore, the greater the number of aromatic compounds in the asphaltene sample, the greater the potential and ability of the oil to change the wettability of the sand from water-wet to oil-wet. However, two samples (B and C) had the same AI value. Since sample C had a higher HPI than sample B, it has a higher ability to establish a hydrogen bond with the sand surface and, as a consequence, has a higher ability for altering surface wettability.

Condensation and substitution are commonly found in complex asphaltene molecules. Equation 8 represents the ratio between the bands associated with aryl ring breathing modes and Ar–H out-of-plane deformation, which is used as an indicator for the amount of substitution and condensation of aromatic rings as the substitution-condensation index (SCI)^108^.

8 $$SCI=\frac{\mathrm{Aryl\,ring\,breathing\,modes}}{{\text{Ar}}-\mathrm{H\,out\,of\,plane\,deformation }}=\frac{{A}_{1650-1520}}{{A}_{900-700}}$$

The results of this indicator are presented in Table 8. The occurrence of substitution in aromatic rings strongly affects the reactivity of aromatic molecules with adjacent molecules. According to this table, the substitution rate in asphaltene samples is based on sequence (9):

9 $${SCI}_{B}<{SCI}_{C}<{SCI}_{D}$$

The amount of substitution and condensation in sample D increases the reactivity of the aromatic rings due to the occurrence of a phenomenon termed electrophilic aromatic substitution. The highest value of adjacent hydrogen substitution in aromatic rings was performed by the hydroxyl group (–OH) and the side alkyl chain group. In organic chemistry, both groups are referred to as ortho and para-directing activators. Groups such as OH, alkyl, and NH_2_ direct an electrophile ion to ortho and para positions on the aromatic ring. The presence of these compounds on the ring creates a several times increase in the reaction speed and reactivity of the compound in comparison to the pure benzene ring. However, it should be noted that the OH impact will be greater than the alkyl paraffin groups.

Sample D has the highest SCI and HPI indices, although the LCI calculated for this sample is much lower compared to the other samples. Therefore, it is expected that the -OH group attached to the central nuclei will be strongly electron-donating, which represents the motion of the π electrons from the substituent to the rings. This will increase the reactivity of the ring to a considerable extent. However, the C=O carbonyl functional group is present as the main substituent in sample A. C=O acts as an electron-withdrawing group and electron attraction from aromatic rings is achieved by resonance, which significantly reduces the reactivity of the aromatic ring.

The aliphaticity index is a quantitative parameter that indicates the presence of an aliphatic functional group in the molecule structure. The equation below illustrates the aliphatic C–H stretching functional group to aromatic-H out-of-plane deformation, which represents the relative amount of hydrogen in the aliphatic group to the aromatic hydrocarbon that can also be calculated via the H-NMR spectrum^108^. The ALI_1_ index has a linear relationship with the aliphatic to aromatic hydrogen ratio when asphaltene particles are considered. The ALI_1_ determines using H-NMR analysis^109^.

10 $${ALI}_{1}=\frac{\mathrm{Aliphatic\,C}-\mathrm{H\,Stretching\,band\,Area}}{{\text{Ar}}-\mathrm{H\,out\,of\,plane\,deformation }}=\frac{{A}_{3000-2800}}{{A}_{900-700}}$$

In addition to the above relation, the A-factor is also an indicator and defined in Eq. 11, showing the relative value of aliphatic groups^110^.

11 $$A-factor=\frac{{A}_{3000-2800}}{{A}_{1650-1520+}{A}_{3000-2800}}$$

The results of both indicators related to aliphatic compounds present in the asphaltene structure are presented in Table 8. The A-factor index has an inverse relationship with the AI. The two asphaltene samples (B and C) with the same AI values also have the same A-factor index values. The A-factor index values have an inverse relationship with the ability of asphaltene to adsorb to the sand surface and alter the wettability. Therefore, paraffinic and naphthenic compounds have a detrimental effect on the reactivity of asphaltene and aromatic-aromatic fractions with the sand surface.

From the perspective of the asphaltene structure, H-bonding and π-bonding through aromatic species will be the main factors involved in the interaction between asphaltene and the sand surface. Therefore, for each asphaltene sample with higher AI and HPI indices, the interaction between that asphaltene and the sand surface would be stronger, and the probability of change in surface wettability would be higher.

Two processes of substitution and condensation of aromatic rings are effective for the severity and weakness of the reactivity of the aromatic rings. If the adjacent hydrogen is replaced by the alkyl- or –OH group, the electron π is shifted from the substituent to the ring, thereby increasing the reactivity of the ring. The presence of functional groups such as carbonyl will have a negative effect on the reactivity of the aromatic ring and will reduce the electron density around the ring.

Paraffinic and naphthenic compounds have a negative effect on the reactivity of asphaltene and the sand surface. The higher the A-factor index in the asphaltene sample, the lower the ability of asphaltene to make the surface oil-wet. The length of the side branches attached to the central nuclei is also decisive. The shorter alkane chain length allows asphaltenes to interact more easily with adjacent molecules.

#### FTIR spectra of sand samples and adsorbed asphaltene

After the injection of oil and paraffinic solvent (n-heptane) at the onset of asphaltene precipitation of any sample, asphaltene deposits form in the sandstone core. Adsorption of polar fractions to the rock surface forces the wettability to trend towards oil-wet. For analysis, thin sections of the cores were taken and thoroughly crushed. The resulting powder comprises sand particles coated with asphaltene. In this section, the FTIR spectrum of sand + asphaltene powder will be studied.

Considering the FTIR spectrum, the composition of the sand can be understood. Quartz is the main constituent of the sand, which is a non-clay mineral and has a main bond of Si–O. Quartz has peaks at different frequency ranges, including the ranges 461–464 cm^−1^ and 509–514 cm^−1^, corresponding to the Si–O asymmetrical bending vibration, the range 690–695 cm^−1^, corresponding to the Si–O symmetrical bending vibration, the ranges 776–780 cm^−1^ and 795–800 cm^−1^, corresponding to the Si–O symmetrical stretching vibration, and the intervals 1080–1085 cm^−1^ and 1169–1171 cm^−1^, corresponding to the Si–O asymmetrical stretching vibration. Feldspar is another important mineral of sand that can be seen in various structures, such as orthoclase and albite. The presence of feldspar in the sand structure is identified by the presence of peaks at 580–583 cm^−1^ [O–Si–(Al)–O bending vibration], and at 640–645 cm^−1^ and 1768–1775 cm^−1^ (Al–O coordination vibration). Clay impurities are also expected in the sand. The peak at a frequency of 1033 cm^−1^ (Si–O characteristic bands of kaolin) indicates the presence of kaolinite in the sand structure^111^. Continuous peaks are present in the structure of kaolinite at 3620 cm^−1^, 3652 cm^−1^, 3670 cm^−1^, and 3697 cm^−1^, corresponding to the O–H stretching group. The peaks corresponding to 3620 cm^−1^ and 3695 cm^−1^ are usually present, but the two peaks at frequencies of 3670 cm^−1^ and 3650 cm^−1^ often disappear from the spectrum.

**Figure 6:**
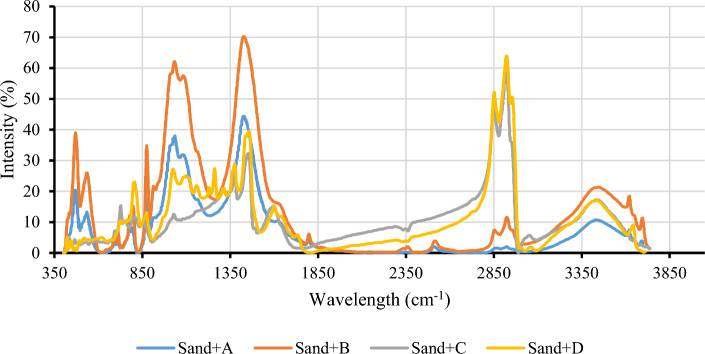
FTIR spectra of sand samples coated with asphaltene.

Calcium carbonate (CaCO_3_) can also be seen in sandstone deposits. The main peak corresponding to calcite is in the 1426–1434 cm^−1^ range, which is specific to calcite and related to CO_3_^2−^ stretching. In the range 874–880 cm^−1^, a peak corresponding to CO_3_^2−^ bending is observed, but its intensity is less than the CO_3_^2−^ stretching peak. The peak at the frequency of 1798–1798 cm^−1^ also corresponds to C–O stretching in carboxylate^112,113^. The appearance of each of the above peaks in the FTIR spectrum of sand coated with asphaltene is related to the sand, indicating a lack of complete coverage of the sand grains by asphaltene. Figure 6 shows the FTIR spectrum of the sand coated with asphaltene. The peaks in the spectrum of each asphaltene-coated sample were compared with peaks in the clean sand spectrum.

Table 10 shows in detail that each peak in the spectrum of sand + asphaltene of each sample is related to sand or asphaltene. According to Table 10, sand coated with asphaltene A (sand + A) and sand coated with asphaltene B (sand + B) show more bonds associated with the sand. Meanwhile, asphaltene A demonstrated the lowest ability to adsorb to the sand surface and produced the smallest change in contact angle.

**Table 10:** The peaks of the FTIR spectrum of sand coated with asphaltene samples (w, m, and s indicate weak, medium, and strong peak intensities, respectively).

Peak/asphaltene sample	Wavenumber (cm^−1^)	Mineral name	Sand + A	Sand + B	Sand + C	Sand + D
O–H str and def	3695	Kaolinite	× (w)	× (w)	–	–
O–H str and def	3620	Kaolinite	× (w)	× (w)	–	× (w)
O–H Str	3436 ± 10	Organic material	× (w)	× (m)	× (m)	× (m)
C–H asym. Str methylene	2924 ± 5	Organic material	× (w)	× (m)	× (s)	× (s)
C–H sym. str methylene	2858 ± 5	Organic material	× (w)	× (m)	× (s)	× (s)
S–H str	2514	Organic material	× (w)	× (w)	–	–
C–O str. carboxylate	1798	Calcite	× (w)	× (w)	–	–
C=O carbonyl	1731	Organic material	–	–	–	× (w)
Si–O	1619	Quartz	× (w)	–	–	–
C=C aromatic	1599	Organic material	–	–	× (m)	× (m)
CH_2_ scissoring mode	1452	Organic material	–	–	× (s)	× (m)
O–H bend	1437	Organic material	–	–	–	× (w)
CO_3_^2−^ str	1429	Calcite	× (s)	× (s)	–	–
CH_3_ umbrella str	1374	Organic material	–	–	× (m)	× (m)
C–O str. Carboxylic acid	1261	Organic material	–	–	–	× (m)
Aromatic C–H in-plane bend	1230	Organic material	–	–	–	× (w)
Si–O asym. str	1165	Quartz	× (w)	–	–	–
Aromatic C–H in-plane bend	1159	Organic material	–	–	–	× (w)
Si–O asym. str	1083	Quartz	× (s)	× (s)	–	–
Si–O	1033	Kaolinite	× (s)	× (w)	–	–
S=O str. Sulfoxide	1030 ± 5	Organic material	–	–	× (w)	× (m)
CO_3_^2−^ bend	876 ± 5	Calcite	× (m)	× (m)	–	× (w)
C–H bend. aromatic	803	Organic material	–	–	× (w)	× (m)
Si–O sym. str	797	Quartz	× (w)	× (m)	–	–
Si–O sym. str	778	Quartz	× (w)	× (m)	–	–
C–H bend. aromatic	745	Organic material	–	–	× (m)	× (w)
CH_2_ rock mode	729 ± 5	Organic material	× (w)	× (w)	× (m)	× (m)
Si–O–Fe	712	Quartz	× (w)	× (w)	–	–
Si–O sym. bend	694	Quartz	× (w)	× (w)	× (w)	–
Si–O asym. bend	536 ± 10	Quartz	× (m)	× (m)	× (w)	× (w)
Si–O asym. bend	471 ± 10	Quartz	× (m)	× (m)	× (w)	× (w)

Two asphaltene samples (A and B) can only be adsorbed to the sand surface through hydrogen bonding and the interaction between the OH group. The only peak corresponding to the polar bond in the two sand + A and sand + B spectra is related to the O–H stretching at a frequency of about 3400 cm^−1^, which is a hydrogen-bonded state. No peaks corresponding to the aromatic groups in the two spectra were observed. The intensity of the OH stretching in the sand + B sample was higher than that of the sand + A sample. As a result, changes in the contact angle due to adsorbing of asphaltene sample B are more intense than sample A.

In the spectrum of the sand + C and sand + D samples, in addition to the peaks associated with the sand constituents, the peaks corresponding to the asphaltene also appeared. The intensity and number of peaks related to sand in the spectrum of these samples were much less than that of sand + A and sand + B. From the peaks associated with the sand, only two peaks at 531 cm^−1^ and 471 cm^−1^ appeared for the sand + C sample. Peaks associated with sand in the sand + D sample were observed at four frequencies of 531 cm^−1^, 471 cm^−1^, 3640 cm^−1^, and 876 cm^−1^, all of which were weak in intensity.

The interaction between the sand surface and asphaltene C occurred through both aromatic compounds and H-bonding. The peaks corresponding to aromatic compounds are at a frequency of 1600 cm^−1^ and within the range of 700–1000 cm^−1^. The appearance of the peaks associated with the hydroxyl group at a frequency of 3440 cm^−1^, and sulfoxide at a frequency of 1030 cm^−1^, suggests that oxygen-containing and sulfur-containing polar compounds play an important role in asphaltene adsorption to the sand surface, respectively.

The interaction between the sand and asphaltenes of sample D is similar to that of asphaltene sample C, through both aromatic compounds and H-bonding. However, the appearance of additional peaks in the FTIR spectrum, which cannot be accounted for by the two mechanisms mentioned, shows that the concentration of adsorbed compounds on the sand surface and the change in wettability are higher compared to the previous samples. In the sand + D spectrum, the peaks at 1600 cm^−1^, 700–1000 cm^−1^, and 1230 cm^−1^ correspond to the C–H aromatic in-plane bending. This is a weak peak in the FTIR spectrum of an aromatic sample, and its appearance represents the high concentrations of aromatics. In addition, in the sand + C sample, only the OH streaking peak was observed from the hydroxyl functional group. In the sand + D spectrum, all possible peaks for the carboxylic acid functional group appeared, including a peak at a frequency of 3400 cm^−1^, corresponding to OH stretching, at a frequency of 1730 cm^−1^, corresponding to C–O stretching, at a frequency of 1430 cm^−1^, corresponding to OH bending, and at a frequency of 1260 cm^−1^, corresponding to the vibration of C–O stretching. Therefore, the carboxylic acid functional group present in the asphaltene sample D structure plays an important role in adsorbing asphaltene to the surface. The claim in this section is consistent with the results of the calculated HPI for each sample.

Peak intensity within the frequency range 2800–3000 cm^−1^, which represents the most intense peak in the pure asphaltene spectrum, can be used as a rule of thumb for determining the surface wettability and contact angle alteration of the sand. In the sand + A, there is almost no peak in the range of 2800–3000 cm^−1^. In sand + B, there are some peaks in this range, but the intensity is not significant. However, very sharp peaks appeared in this range in the sand + C and sand + D samples.

### Characterisation by zeta potential and elemental analysis

Four asphaltene samples extracted from crude oil by ASTM-D6560 (IP143 standard method) were placed for elemental analysis, with the results demonstrated in Table 11. Asphaltene is a heavy hydrocarbon fraction, consisting mainly of hydrogen and carbon, and is the most polar fraction of crude oil, which also contains heteroatom elements such as sulfur, nitrogen, and oxygen (SNO) in its molecular structure^114^.

**Table 11:** Elemental analysis results for four asphaltene samples extracted from crude oil.

	Asphaltene Samples			
	A	B	C	D
C	82.31	82.61	83.59	83.09
H	10.11	9.53	6.81	10.11
S	3.59	3.66	5.15	4.31
N	2.77	3.08	1.21	1.32
O	1.22	1.12	3.24	1.17

According to Table 11, the heteroatoms existing in the asphaltene structure do not have a direct relationship with the ability of asphaltene to change the surface wettability of sand. This is due to the fact that all of the asphaltene fractions do not directly affect surface wettability. Previous studies have shown that asphaltene in the presence of sand is divided into four sub-fractions including bulk, normal-adsorbed, hard-adsorbed, and irreversible-adsorbed^49^. The adsorbed sub-fractions play a significant role in changing the surface wettability from water-wetness to oil-wetness. These fractions adsorb to the surface through rock-asphaltene interaction and create a layer of asphaltene as a mono-layer at the surface. However, with the formation of a mono-layer on the surface of the rock, asphaltene molecules adsorb onto the surface through asphaltene-asphaltene interaction. These fractions have no significant effect on wettability and only affect the porosity and permeability of the surface. Also, some of the elements in asphaltene are adsorbed depending on the surface of the rock (calcite or sandstone) and observed in adsorbed sub-fractions, and a large portion of the compounds containing SNO elements will be present in the bulk phase^53^.

The adsorption of asphaltene fractions to the surface of sand powder in the absence of dynamic flow has been studied^53^. Sulfur is a two-dimensional element, which is mainly observed in bulk fractions and also observed in adsorbed fractions. According to Table 11, asphaltene samples C and D contain the highest amounts of sulfur and have the greatest ability to make the sand surface oil-wet. Sulfur has a higher contribution to the adsorption of these samples, as sulfur occurs naturally in asphaltene. Two functional groups of thiophene and sulfide are the main sulfur functional groups in asphaltene, which are acidic and capable of acid–base interaction with the sand surface. There are no compounds like sulfate and pyrite in asphaltene. It should be noted that the amount of sulfur in sample C is higher than that of sample D, while less change in surface contact angle is observed in sample C. This is due to the fact that sulfur is often present as reduced sulfur in the thiophene structure. Over time, due to sulfide oxidation, the oxidized sulfoxide functional group increases in abundance within the asphaltene structure. Referring to the literature, the sulfoxide present in the asphaltene structure is not primarily bound to aromatic compounds. This leads to a reduction in its capacity to adsorb to the sand surface and it is therefore only observed in bulk fractions^115^ since the aromatic compounds exhibit the highest tendency to adsorb to the surface of the sand.

Another heteroatom in the molecular structure of asphaltene is Nitrogen, which is visible in two functional groups pyrrolic components and pyridinic components. Nitrogen lone pair electrons are part of the aromatic ring in the pyrrole aromatic group. For this reason, they have a low ability to form a new bond with the proton that will eventually give the asphaltene a weak basic feature^116^. A basic group with similar properties to tertiary amine is the pyridine functional group^117^. The saturated amine functional group is not normally present in asphaltene^118^. Hence, it can be concluded that the presence of nitrogen in asphaltene is mainly in the form of basic groups. With the basis of Table 11, the nitrogen content is higher in both asphaltene samples A and B. The presence of nitrogen in the asphaltene structure decreases its tendency for surface adsorption. Nitrogen can only increase the adsorption of asphaltene to the surface if the surface of the sand contains significant amounts of clay^119^. According to the literature, the nitrogen content plays a key role on the asphaltene adsorption on quartz surfaces, if the asphaltene has high polarity^16,120^. It’s worth noting that a substantial portion of the minerals found in sandstone consists of quartz^26,121^. Consequently, the adsorption of asphaltene particles onto sandstone surfaces tends to increase as both the polarity of asphaltene and its nitrogen content increase. In simpler terms, higher nitrogen content can enhance the adsorption of asphaltene onto sandstone when the asphaltene exhibits high polarity.

One of the most substantial elements in the asphaltene structure is oxygen. About 40–70% of the asphaltene structure consists in the form of hydroxyl groups, which according to their position in the molecule, are capable of acetylation^97^. The hydroxyl group, along with the carbonyl group, forms the carboxylic acid group, which is a weak acid^122^. Therefore, sulfur and oxygen together create the acidity of asphaltenes. Sequence (12) shows the total oxygen and sulfur ($\vartheta$) in each sample of asphaltene:

12 $${\vartheta }_{A}(4.78\%)\approx {\vartheta }_{B}(4.81\%)<{\vartheta }_{D}(5.48\%)<{\vartheta }_{C}(6.36\%)$$

Firstly, the sequence above shows that two samples with a higher $\vartheta$ will have a stronger interaction with the surface. Secondly, polar interaction is not necessarily the main mechanism for asphaltene adsorption to the sand surface. Asphaltene sample C has a higher acidic component than sample D, but it does not necessarily alter the surface wettability more than sample D.

**Figure 7:**
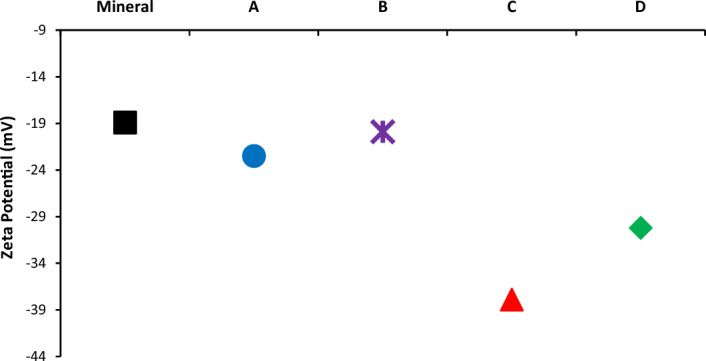
The zeta potential values for clean sand and sand covered with asphaltene.

In order to investigate the above issues, the sand sample covered with each asphaltene sample was analyzed by the zeta potential measurement. The test results are shown in Fig. 7. Clean mineral sand without any surface asphaltene showed no negative zeta potential (about − 19 mv). The surface of the dry sand is composed of siloxane and silanol groups, both of which have an H-bonded hydroxyl group and a free hydroxyl group^122^. The silanol functional group contains hydroxyl, and its ability to form hydrogen bonds is greater than that of water molecules. Their presence in the sand has given it a high acidity, as some studies have shown an acidity constant of sand of between 5.6 and 8.5 in the presence of silanol^123,124^.

Therefore, H-bonding is one of the most significant mechanisms for adsorption between the rock surface and the asphaltene particle. Based on the discussion of the FTIR results, asphaltene sample D has the highest potential for hydrogen bonding and π-bonding with the surface of the rock and therefore has the greatest change in surface wettability. However, Fig. 7 shows that the surface electrostatic properties of the sand, despite similarities with surface wettability, have a fundamental difference. Sequence (13) shows the zeta potential values of the sand surface after the adsorption of each asphaltene sample:

13 $${\xi }_{C}<{\xi }_{D}<{\xi }_{A}<{\xi }_{B}$$

The adsorption of active asphaltene substances to the sand surface is a significant phenomenon that can change the surface charge. Normally in a sandstone sample, the surface charge of a solid particle is a function of the potential determining ions concentration in the environment. These ions are the same as H^+^ and OH^−^. Therefore, the presence of the most acidic compounds in the asphaltene structure adsorbed to the surface of the rock will induce a more negative surface zeta potential. Sequence (12) showed that asphaltene sample C probably contains the highest amount of acidic compounds in its molecular structure. For this reason, the adsorption of asphaltene sample C to the sand surface has produced the highest level of surface charge change. However, this does not necessarily mean that the surface is more oil-wet. Sample D has higher levels of the AI and HPI indices compared to the other asphaltene samples. The presence of aromatic fractions in the asphaltene will not change the surface charge, but is expected to change the surface wettability to be significantly oil-wet. Changing the wettability of the surface, in addition to acid–base interaction and H-bonding, is highly dependent on the π-bonding between asphaltene and the sand surface^125,126^.

Asphaltene sample B, according to the contact angle results, adsorbs to the sand surface and pushes the surface towards being oil-wet. However, as shown in Fig. 7, adsorption of this asphaltene sample did not change the charge of the sand surface, due to the presence of basic compounds such as nitrogen. Table 11 showed earlier that sample B had the highest nitrogen content among the four samples. Due to the acid–base interaction mechanism, the basic compounds adsorb to the acidic surface of the sand, however, the zeta-potential pushes to more positive values. This phenomenon is another reason for the difference between the two concepts of wettability and surface charge.

### Characterisation by SEM imaging and elemental mapping

**Figure 8:**
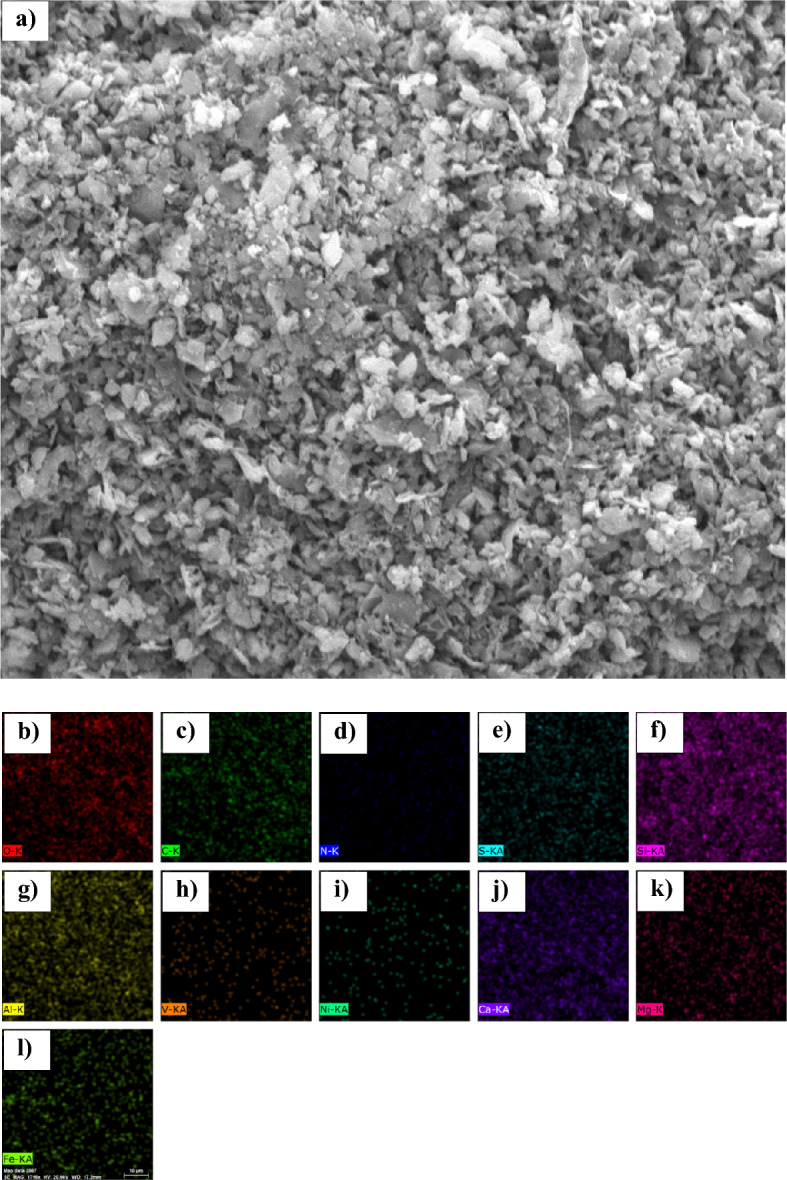
(**a**) SEM image and energy dispersive X-ray (EDX) elemental maps for (**b**) Oxygen, (**c**) Carbon, (**d**) Nitrogen, (**e**) Sulfur, (**f**) Silicon, (**g**) Aluminium, (**h**) Vanadium, (**i**) Nickel, (**j**) Calcium, (**k**) Magnecium, and (**l**) Fe (Iron) on the cross section of sandstone in the presence of asphaltene sample A.

**Figure 9:**
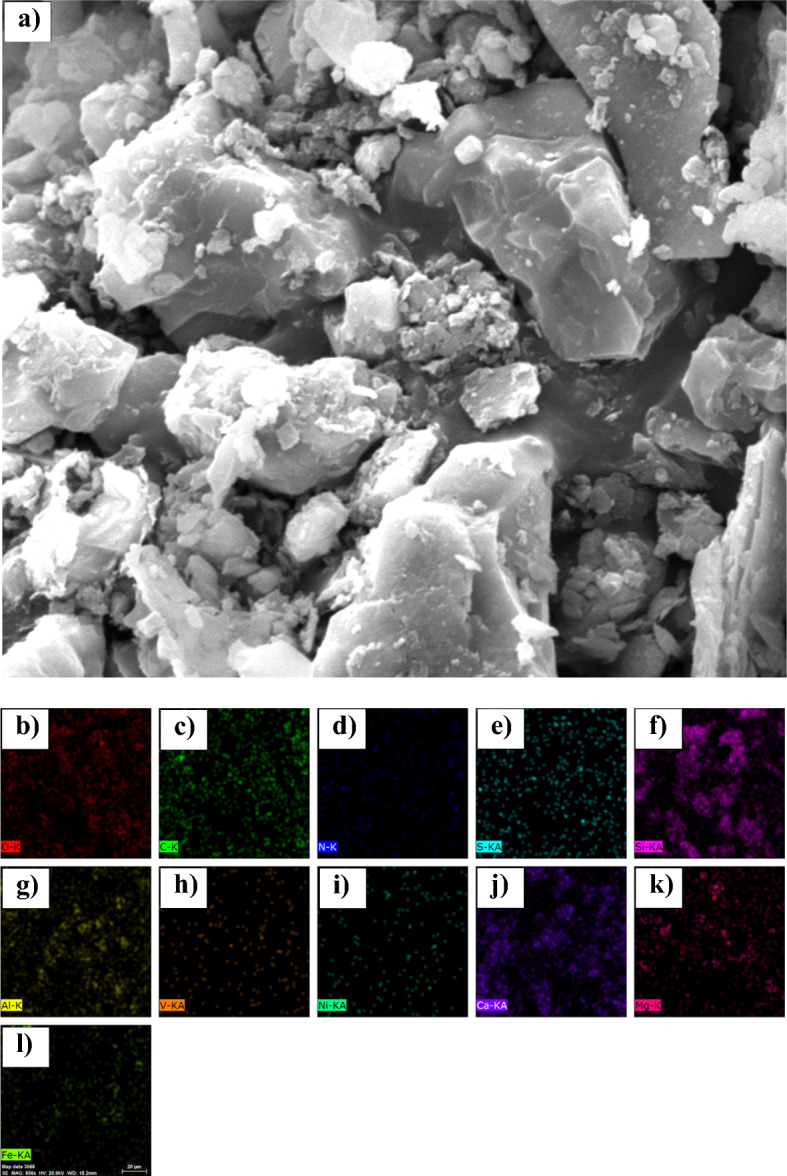
(**a**) SEM image and EDX elemental maps for (**b**) Oxygen, (**c**) Carbon, (**d**) Nitrogen, (**e**) Sulfur, (**f**) Silicon, (**g**) Aluminium, (**h**) Vanadium, (**i**) Nickel, (**j**) Calcium, (**k**) Magnecium, and (**l**) Fe (Iron) on the cross section of sandstone in the presence of asphaltene sample B.

**Figure 10:**
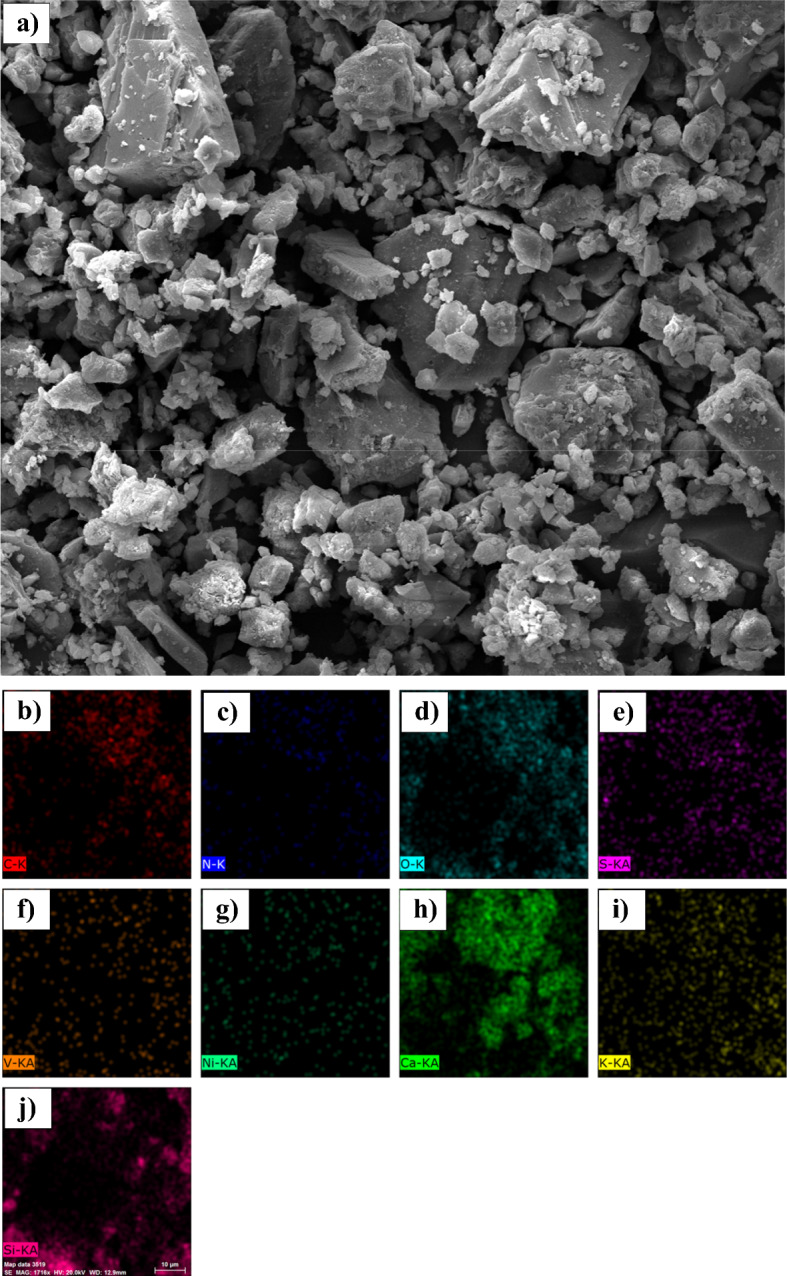
(**a**) SEM image and EDX elemental maps for (**b**) Carbon, (**c**) Nitrogen, (**d**) Oxygen, (**e**) Sulfur, (**f**) Vanadium, (**g**) Nickel, (**h**) Calcium, (**i**) Potasum, (**j**) Silicon on the cross section of sandstone in the presence of asphaltene sample C.

**Figure 11:**
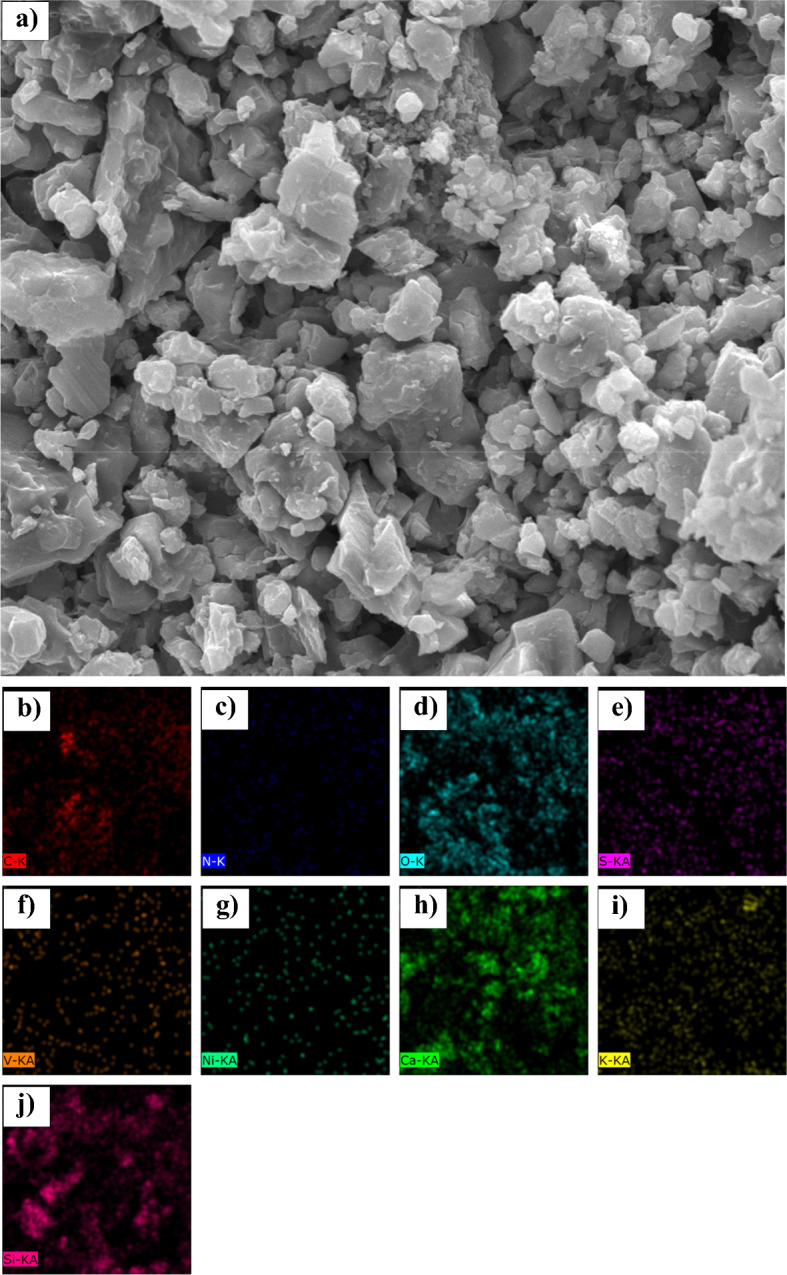
(**a**) SEM image and EDX elemental maps for (**b**) Carbon, (**c**) Nitrogen, (**d**) Oxygen, (**e**) Sulfur, (**f**) Vanadium, (**g**) Nickel, (**h**) Calcium, (**i**) Potasum, (**j**) Silicon on the cross section of sandstone in the presence of asphaltene sample D.

Figures 8, 9, 10, 11 show the overall structure of the sand surface covered with asphaltene samples. The energy-dispersive X-ray spectroscopy (EDX) analysis (Table 12) was also performed for elemental mapping and to determine the distribution of elements on the sand surface after asphaltene precipitation.

**Table 12:** The results of elemental mapping of sand covered with asphaltene.

Asphaltene sample	A (%)	B (%)	C (%)	D (%)
O	60.18	46.86	42.64	37.98
S	0.69	0.54	2.19	0.84
N	0.98	1.46	0.00	0.79
C	8.23	21.97	39.57	41.56
Ca	10.38	10.00	5.28	5.43
Si	12.03	10.94	7.07	8.21
Fe	2.00	0.88	0.83	0.92
Al	3.82	3.33	1.62	2.99
K	1.00	1.59	–	–
Mg	0.61	1.19	0.66	1.24
V	0.00	0.05	0.03	0.06
Ni	0.00	0.07	0.12	0.00
Na	–	0.96	–	–

According to the results presented in the contact angle section, it was found that asphaltene samples A and B had the lowest ability to change the surface wettability, and samples C and D exhibited the greatest change in contact angle. The amount of oxygen resulting from mapping the coated sand surface is based on the following sequence:

14 $${O\%}_{D}<{O\%}_{C}<{O\%}_{B}<{O\%}_{A}$$

The oxygen content of the surface decreases with asphaltene adsorption. In other words, by adsorption of asphaltene sample D (which produces the greatest change in wettability), the amount of oxygen on the surface of the sand is less than that of sample A. This is also due to the hydrocarbon nature of asphaltene. However, despite its polarity, asphaltene has a lower oxygen content than quartz (SiO_2_), calcite (CaCO_3_), and clay minerals. The greater the surface area covered by asphaltene, the lower the amount of oxygen observed by elemental mapping.

Moreover, increasing the asphaltene adsorption to the surface (oil-wetting of the surface) will increase the percentage of carbon derived from elemental mapping. Sequence (15) shows a comparison of the amount of carbon for sand coated with the four samples of asphaltene:

15 $${C\%}_{D}>{C\%}_{C}>{C\%}_{B}>{C\%}_{A}$$

The contact angle for the sand samples was very close after the adsorption of asphaltene samples C and D, with more oil-wetting of sample D compared to C. This is also seen in the carbon content of the sand surface. After asphaltene adsorption, the carbon content of samples C and D was 41.56% and 39.57%, respectively. This also demonstrates that the aromatic asphaltene fractions play a major role in adsorbing on the sand surface and changing the wettability, since carbon is the main component of the aromatic compounds, and the trend of the wettability of sand is completely different from the changes in carbon surface area after asphaltene adsorption.

As noted earlier and based on the zeta potential results, presence of nitrogen in asphaltene is mainly in the form of basic groups (pyrrolic, and pyridinic groups). However, sulfur mostly presents in asphaltene structure in forms of acidic groups (thiophene (C_4_H_4_S), and sulfide (S^2−^)). Presence of sulfur and nitrogen changes the electrostatic properties of asphaltene and the surface through acid–base reactions with the acidic and basic components present in the fluid phase. However, the asphaltene adsorbed to the rock surface does not necessarily have high nitrogen and sulfur contents. This claim is approved here. No significant relationship was found between the contact angle of the sand and the amount of nitrogen and sulfur present on the surface after the adsorption of asphaltene.

This occurred during the adsorption of asphaltene samples A and B to the surface. Also, the high sulfur content in asphaltene powder increases the sulfur content of the sand surface after asphaltene adsorption. This will reduce the electrical potential of the rock surface, but will not necessarily make the surface oil-wet.

In addition to quartz, calcite and clay minerals are also found in the sandstone structure. The presence of metals such as sodium, potassium, iron, and aluminum in Table 12 indicates the presence of clay minerals in the rock structure. Similar to the structure of phyllosilicates, clay minerals consist of Fe_2_O_3_, SiO_2_, and Al_2_O_3_ sheets. Aluminum and iron are found on the surface of clay minerals. Due to the composition of the tetrahedral and octahedral sheets, the sheets can have either a neutral or negative charge, requiring a cation such as potassium (K^+^) or sodium (Na^+^) to attain equilibrium. The presence of cations with a positive charge in Table 12 also suggests the presence of clay minerals^127,128^.

The percentage of minerals corresponding to rock minerals in the mapping is related to the degree of wettability. For example, according to Table 12, the distribution of silicate elements on the sand surface follows the sequence (16):

16 $${Si\%}_{C}<{Si\%}_{D}<{Si\%}_{B}<{Si\%}_{A}$$

Silicate is observed in the structure of quartz and clay minerals. Lower adsorption of asphaltene to the sand surface and, consequently, keeping the surface intact, results in a greater amount of silicates in elemental mapping. Calcium is another important mineral in sandstone formations. The amount of calcite observed at the surface after the adsorption of the polar fractions of asphaltene samples is consistent with sequence (17):

17 $${Ca\%}_{C}<{Ca\%}_{D}<{Ca\%}_{B}<{Ca\%}_{A}$$

The trend for calcium is exactly the same as that of silicates. The above process was also observed for other metals in the rock structure. The presence of metals on the sand surface has an effect on the surface zeta potential. Asphaltene samples A and B are less adsorbed onto the sand surface, therefore, according to Table 12, the amounts of metal cations present on the sand surface in these two samples are significant. This has the potential to make the surface zeta potential more positive compared to the adsorption of asphaltene samples C and D. This claim is also related to the results of previous studies^129,130^. The metal cations contained in the clay structure result in less reduction of the zeta potential. Their value depends on two factors, including (1) valency (which depends on the amount of cation charge and, with increasing positive charge, the surface zeta potential will be more positive), and (2) intrinsic cation radius (which will induce a positive zeta potential by increasing the inner radius)^129,130^.

Trace amounts of vanadium and nickel were also observed in the elemental mapping of the sand surface, but these metals do not play a significant role in changing the properties (such as wettability) of the sand surface.

We conclude that carbon is the main indicator of changing wettability in the elemental mapping of the sand surface. The higher the carbon content, the greater the change in wettability and trend towards oil-wetting of the surface. Oxygen content, seen both on the sand surface and in asphaltene, is not a good criterion for assessing the properties of sand as a result of asphaltene precipitation. Nitrogen and sulfur are observed in the basic and acidic functional groups, respectively, and their presence on the sand surface changes the electrostatic properties. A sand surface coated with asphaltene will reduce the percentage of metal cations appearing on the surface.

## Conclusions

Precipitation of asphaltene in porous media leads to decrease in permeability of rock and consequently reduce the oil recovery. Molecular characteristics of asphaltene particles and minerology of rock surface plays a great role in the intensity of asphaltene adsorption. In this study, the effects of four different asphaltene samples on the wettability of sandstone reservoir are investigated. The contact angle and relative permeability are determined, to evaluate the wettability alteration comprehensively. The value of contact angle belong to asphaltene sample A, B, C, and D with sandstone rock was equal to 115°, 123°, 139°, and 141°, respectively. By comparing the results obtained from contact angle and EDX analysis, the contact angle increases from 115° to 141° by increase in adsorption of carbon on the sand surface from 8.23% to 41.56%. The FTIR spectroscopy results show that hydrogen-bonding, π-bonding, and sulfur-containing compounds such as sulfoxide improve asphaltene adsorption onto the sand surface. The higher the aromaticity index (AI) and hydrogen potential index (HPI) obtained from FTIR analysis, the greater the ability of asphaltene to change wettability. According to zeta potential analysis, adsorption of heteroatoms (N, O, and S) and surface active components present in asphaltene would make the surface charge of the sand more negative. However, this does not necessarily mean that the surface is oil-wet, and two concepts of wettability and surface charge may be differentiated. Elemental mapping demonstrated that the presence of carbon on the surface was the main indication of asphaltene adsorption and wettability alteration in comparison with adsorption of heteroatoms. Novel findings of the present study show that the presence of nitrogen/sulfur-containing functional groups on the sand surface changes the electrostatic properties, as a sand surface coated with asphaltene will reduce the percentage of metal cations appearing on the surface.

In the next study, an attempt will be made to investigate the effect of temperature on the interaction between rock and fluid and the wettability alteration process.

## Data Availability

All data generated or analysed during this study are included in this published article.
